# Long Noncoding RNA LINC02249 Is a Prognostic Biomarker and Correlates with Immunosuppressive Microenvironment in Skin Cutaneous Melanoma

**DOI:** 10.1155/2022/2054901

**Published:** 2022-09-07

**Authors:** Maotao Du, Liang Han, Pan Shen, Dengyan Wu, Shenghao Tu

**Affiliations:** ^1^Department of Integrated Chinese Traditional and Western Medicine, Tongji Hospital, Tongji Medical College, Huazhong University of Science and Technology, Wuhan, China; ^2^Department of Dermatology and Plastic Surgery, The Second Affiliated Hospital of Chongqing Medical University, Chongqing 400010, China

## Abstract

Skin cutaneous melanoma (SKCM) is one of the most aggressive and life-threatening tumors. It has a high incidence rate, as well as significant metastasis and fatality rates. To successfully treat SKCM and to increase the overall survival rate, early identification and risk stratification are both absolutely necessary. Long noncoding RNAs (lncRNAs) play a significant regulatory role in a variety of cancers. However, the expression and function of many lncRNAs have not been investigated. We evaluated the expression profile of the long noncoding RNA LINC02249 (LINC02249) in pan-cancers by using data on gene expression obtained from TCGA and GTEx. The biological function of LINC02249 was determined by gene ontology (GO) and Kyoto Encyclopedia of Genes and Genomes (KEGG). The prognostic value of LINC02249 expression in SKCM patients was statistically analyzed. Besides, the ssGSEA approach was utilized in order to investigate the degree to which LINC02249 expression is correlated with tumor immune infiltration. In this study, the expression of LINC02249 was found to be abnormally high in a variety of tumors, according to our findings. When compared with nontumor specimens, the level of expression of LINC02249 was shown to be significantly elevated in SKCM samples. GO and KEGG assays revealed LINC02249 may be involved in tumor progression. High expression of LINC02249 was associated with shorter overall survival and disease-specific survival of SKCM patients. More importantly, multivariate methods revealed that LINC02249 expression was an independent prognostic factor for SKCM cases. Using ssGSEA, we found that the expression of LINC02249 was negatively associated with different tumor-infiltrating immune cells, especially aDC, Treg, and macrophages. Overall, our findings suggested that LINC02249 can serve as a novel biomarker to predict the prognosis and immune infiltration in SKCM.

## 1. Introduction

Skin cutaneous melanoma (SKCM) is a malignant transformation of melanocytes derived from neural crest stem cells [1]. Over the course of the last ten years, the prevalence of SKCM has skyrocketed across the globe [2]. Despite the fact that SKCM only accounts for about 5 percent of all skin tumors, it is responsible for more than 75 percent of deaths that are caused by skin tumors [3, 4]. In addition, the majority of people diagnosed with melanoma experience relapses or do not respond to therapies because of toxicity, intrinsic drug resistance, and other factors that are not fully understood [5, 6]. Internal heterogeneity is shown by the molecular properties of SKCM; this is the primary factor that prevents customized treatment and is the primary factor in determining drug resistance [7, 8]. The dissatisfied prognosis of SKCM has not changed considerably despite the fact that numerous treatments, including phototherapy, chemotherapy, immunotherapy, local resection, and radiotherapy, have been used in SKCM patients [9]. Besides, early diagnosis of SKCM is still a huge challenge. Thus, it is of the utmost need to find novel biomarkers that are effective in detecting, diagnosing, and predicting the prognosis of GC.

Long noncoding RNA (lncRNA) is a class of noncoding RNA greater than 200 nucleotides in length [10]. Increasing studies have shown that lncRNAs play key roles in the processes of chromosomal silencing, chromatin epigenetic modification, gene transcription, protein translation, and protein localization [11]. It is important to highlight that abnormal regulation of lncRNAs has been linked to the development and progression in a variety of human cancers [12, 13]. Several lncRNAs, such as lncRNA HCP5, lncRNA TEX41, and lncRNA TTN-AS1, play important roles in the growth of malignant cells in SKCM [14–16]. In addition, there are a growing number of studies that point to the fact that abnormally expressed lncRNAs have been utilized as possible biomarkers for the diagnosis and prognosis of cancer [17, 18]. lncRNA MALAT1, which is a 6.5-kilobase pair long noncoding RNA, has been discovered to be involved in multiple steps in the development of tumors. It also demonstrated diagnostic and prognostic significance in several neoplasms, including melanoma, lung cancer, breast cancer, glioma, and prostate cancer [19–23]. As a whole, the emerging linkages between noncoding RNAs and cancers have heralded the possibility that lncRNAs may serve either as diagnostic biomarkers or therapeutic targets for SKCM.

By exploiting public databases, we find that lncRNA LINC02249, mapped to chromosome 15q13.2, exhibited a dysregulated level in most types of tumors. To date, the potential function of LINC02249 has not been investigated. Our research aimed to clarify the associations between LINC02249 and SKCM using TCGA datasets.

## 2. Materials and Methods

### 2.1. Data Download and Preprocessing

TCGA, which acts as a public repository used to analyze high-throughput microarray data, was applied to obtain the gene expression profile as well as the clinical information of SKCM patients. TCGA datasets included 471 SKCM tissues and 1 nontumor tissue. Subsequent processing excluded cases with insufficient or complete information regarding age, overall survival time, and TNM stage. Because the TCGA database does not contain any samples of normal tissue, controls were derived from the genotype-tissue expression (GTEx) database instead. The full names of cancers in TCGA are shown in Table S1.

### 2.2. Screening of Differentially Expressed Genes (DEGs)

Finding DEGs between two groups (high LINC02249 expression group and low LINC02249 expression group), which were characterized by the median expression level of LINC02249, was accomplished by applying the RNA-seq data obtained from the TCGA-SKCM. The “DESeq2” R package was used to screen for DEGS, while the “heatmap” R package was utilized to plot heatmaps in two different cohorts. Significant upregulation of DEGs was defined as having a *p* value of less than 0.05 and a log FC value more than 1.5; significant downregulation of DEGS was defined as having a *p* value of less than 0.05 and a log FC value less than 1.5.

### 2.3. Kyoto Encyclopedia of Genes and Genomes (KEGG) and Gene Ontology (GO) Pathway Analysis

The GO analysis is broken down into three sections, which are the cellular component, the molecular function, and the biological process. These sections each explain the biological function of a specific gene from their own unique perspective. KEGG is an analysis tool that is used to figure out which biological pathways' particular genes are significantly overrepresented in. After that, we carry out a study of GO and KEGG pathways based on DEGS with low expression versus high expression of LINC02249.

### 2.4. Estimation of Stromal and Immune Scores

The ESTIMATE technique was used to standardize the expression matrix in order to facilitate accurate estimation of the stromal and immunological scores [24]. A single-sample gene set enrichment analysis was performed, and the results were used to construct stromal and immunological scores. The ESTIMATE score was derived using these scores; thus they should be considered reliable.

### 2.5. Tumor Infiltration Analysis

The XIANTAO platform (https://www.xiantao.love/) was used to conduct an analysis of the immune infiltration profiles of the tumors. A total of 24 immunological markers were utilized in order to differentiate between the various immunocytes. Using the single-sample generalized estimating equations analysis (ssGSEA) method, we were able to determine the Spearman correlations between immunocyte markers and LINC02249 expression levels.

### 2.6. Statistical Analysis

All statistical analyses were performed with R software 3.5.3. The unpaired *t*-test was utilized to investigate the possibility of differential expression of LINC02249 in cancer tissues in comparison to nontumor specimens. We used chi-square and *t*-tests to investigate whether or not there was a correlation between the levels of LINC02249 expression and the clinicopathological characteristics. The Kaplan–Meier survival curves were built to analyze survival differences between the high‐LINC02249 group and low-LINC02249 expression group. Univariate and multivariate assays were developed in order to assess the HRs of clinical elements. A two-tailed *p* < 0.05 was considered to indicate a statistically significant difference.

## 3. Results

### 3.1. LINC02249 Expression Analysis in Pan-Cancer

**Figure 1:**
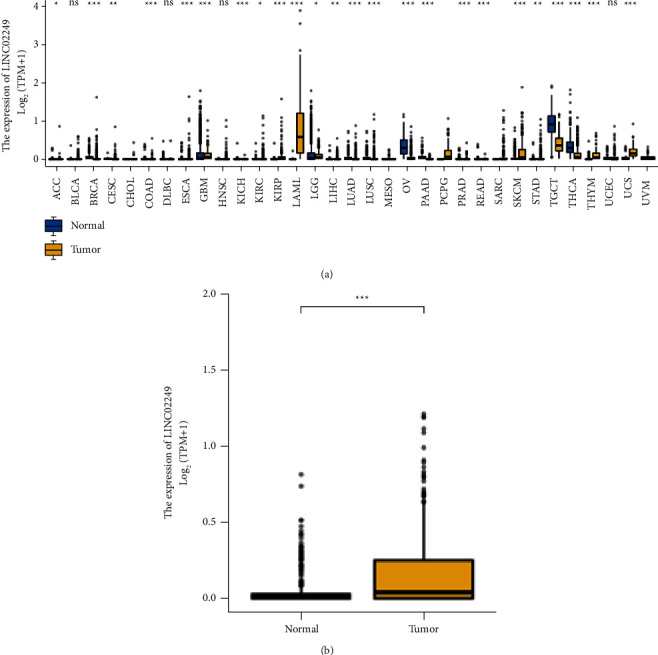
Expression assays for LINC02249 in multiple tumors. (a) LINC02249 expression in pan-cancer (^*∗*^*p* < 0.05; ^*∗∗*^*p* <  0.001; and ^*∗∗∗*^*p* < 0.0001). (b) LINC02249 expression was distinctly increased in SKCM specimens compared with nontumor specimens. ^*∗∗*^^*∗*^*p* <  0.001.

Firstly, we examined the expression of LINC02249 in TCGA and GTEx pan-cancer datasets. According to the findings, a significant amount of LINC02249 expression was found in five different tumors: GBM, KIRP, LAML, LGG, SKCM, and THYM. In comparison, low LINC02249 expression was observed in 18 tumors: ACC, BLCA, CESC, COAD, ESCA, KICH, KIRC, LIHC, LUAD, LUSC, OV, PAAD, PRAD, READ, STAD, TGCT, THCA,and UCEC (Figure 1(a)). Our findings suggested that LINC02249 expression was distinctly increased in most types of tumors, suggesting it as an oncogene in tumors. However, high LINC02249 expression was observed in five tumors, especially in SKCM (Figure 1(b)). Our findings suggested LINC02249 may exhibit a different role based on specific tumor types.

### 3.2. KEGG and GO Enrichment Analyses of DEGs

**Figure 2:**
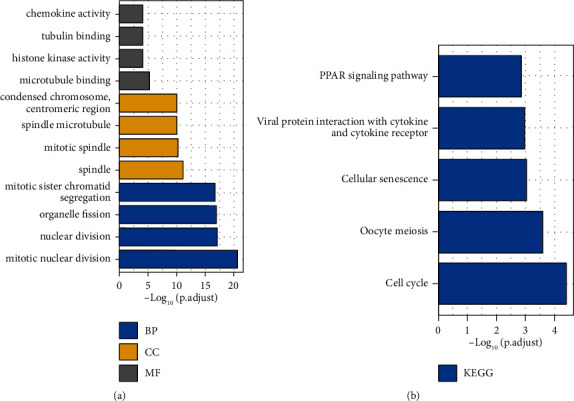
GO terms and KEGG pathway enrichment analysis of LINC02249. (a) GO terms and (b) KEGG pathway enrichment analysis of DEGs between the high LINC02249 expression group and low LINC02249 expression group.

We analyzed the DEGs between LINC02249 high and low expression subgroups and identified 135 DEGs. To study the possible effects of DEGs on tumor progression, we carried out GO enrichment analyses, including biological processes (BP), molecular functions (MF), and cellular components (CC). We observed that changes in MF of DEGs were distinctly enriched in the chemokine activity, tubulin binding, histone kinase activity, and microtubule binding. For CC, DEGs were largely enriched in microtubule binding, spindle microtubule, mitotic spindle, and spindle. Within the BP category, mitotic sister chromatid segregation, organelle fission, nuclear division, and mitotic nuclear division were predominant (Figure 2(a)). KEGG enrichment analysis showed that PPAR signaling pathway, viral protein interaction with cytokine and cytokine receptor, cellular senescence, oocyte meiosis, and ell cycle were significantly enriched in DEGs (Figure 2(b)).

### 3.3. The Clinical Significance and Prognostic Value of LINC02249 in SKCM Patients

**Figure 3:**
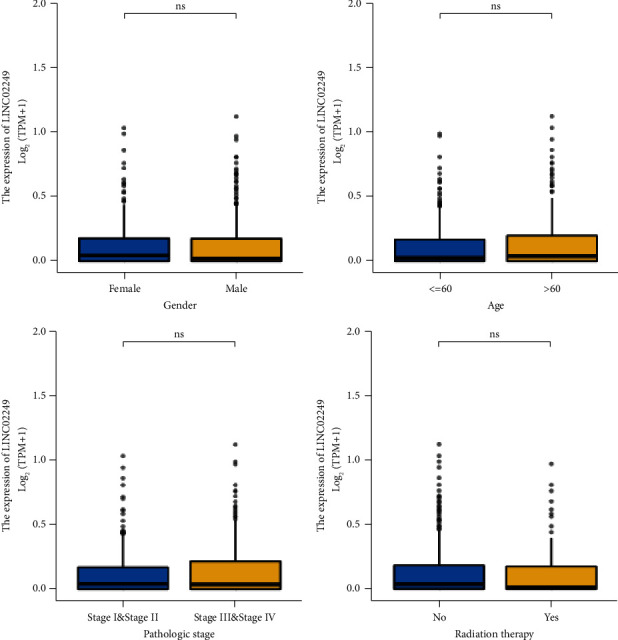
The associations between LINC02249 expression and several clinical factors, including gender, age, pathologic stage, and radiation therapy. ns: no statistical significance.

**Figure 4:**
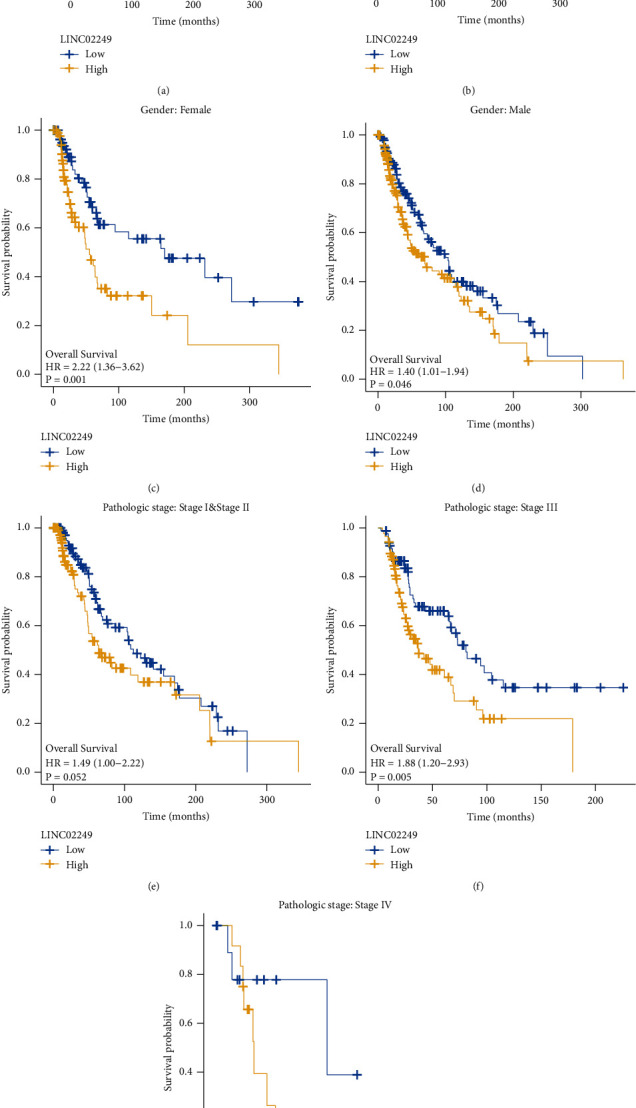
Kaplan–Meier survival curves for SKCM patients in TCGA dataset. (a, b) The overall survival and disease-specific survival rates of the high LINC02249 expression group and low LINC02249 expression group. (d–g) Stratified analyses of clinicopathological factors in SKCM: (c) female, (d) male, (e) pathologic stage (I-II), (f) pathologic stage (I-II), and (g) pathologic stage (IV).

We analyzed the relationship between the LINC02249 expression level and the clinicopathological factors of the SKCM patients in order to elucidate the underlying function of LINC02249 in the development of SKCM. This was done so that we could better understand how LINC02249 contributed to the progression of SKCM. However, there was no significant association with the normal factors (Figure 3 and Table 1). To further investigate the correlation between LINC02249 expression and long-term survivals of SKCM patients, we used Kaplan–Meier survival and log-rank analysis. We found that patients with high LINC02249 expressions showed a shorter overall survival (Figure 4(a)) and disease-specific survival (Figure 4(b)) than those with low LINC02249 expression. Moreover, we performed subgroup analysis. Interestingly, we found that LINC02249 was more suitable for the prediction of the clinical outcomes of female patients than male patients (*p*=0.001*vs*. *p*=0.046, Figures 4(c) and 4(d)). On the other hand, we observed it was better that LINC02249 was used to predict the overall survival of SKCM patients with advanced stage (Figures 4(e)–4(g)). Finally, we performed Cox proportional hazards regression analysis to explore the effects of LINC02249 and clinicopathological factors on patient survival. The univariate analysis demonstrated that pathologic stage, age, and LINC02249 expression were significantly associated with overall survival (Table 2) and disease-specific survival (Table 3) of SKCM patients. More importantly, multivariate assays showed that LINC02249 was an independent prognostic factor for overall survival (Table 2) and disease-specific survival (Table 3) of SKCM patients.

### 3.4. Relationship of Estimate-Stromal-Immune Scores and LINC02249 Expression

**Figure 5:**
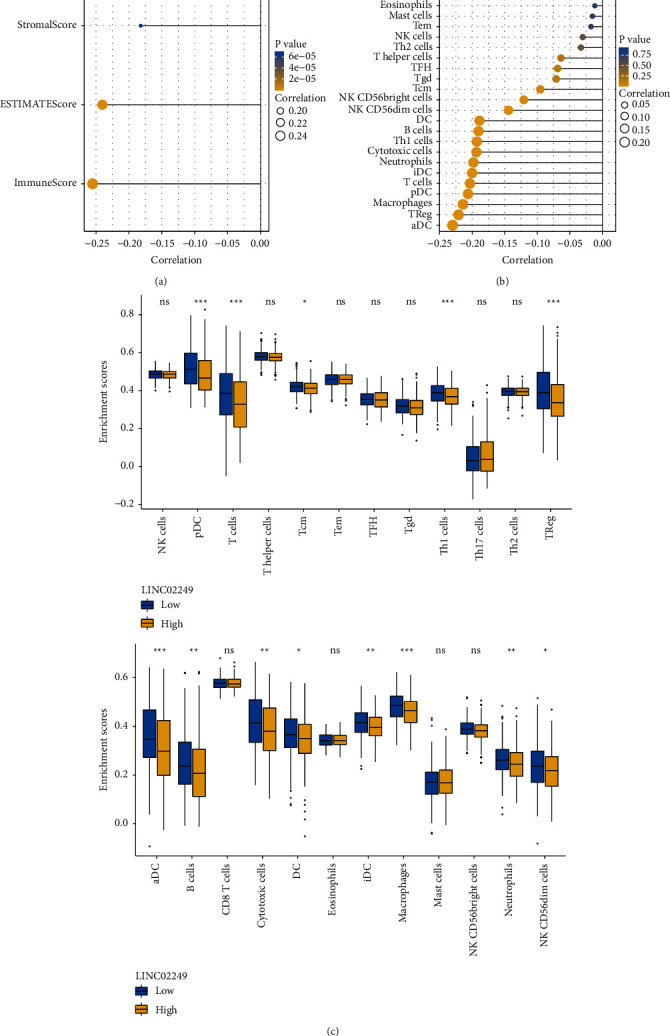
Immune infiltration analysis of LINC02249. (a) The association between LINC02249 expression and immune and stromal scores by the use of ESTIMATE algorithm. (b, c) In order to measure the difference in immune infiltration between patients with low and high LINC02249 levels, the ssGSEA algorithm was applied.

Subsequently, using the estimate package in R, we compared the immunological and stromal scores of each of these individuals. The subsequent step was to collect data from 471 patients who had complete immune and stromal scores. We found the expression of LINC02249 was distinctly negatively associated with stromal scores, immune scores, and estimate scores (Figure 5(a)).

### 3.5. LINC02249 Expression Correlates with Immune Cell Infiltration in SKCM Tissues

The degree of immune infiltration into the microenvironment of the tumor was an important component in determining both the effectiveness of anticancer treatment and the final prognosis of the patient. In SKCM tissues, we investigated whether or not there was a link between the expression of LINC02249 and immune infiltration profiles. The findings demonstrated that out of the 24 different types of infiltrating immune cells, 11 of those types displayed a strong inverse correlation with the following: NK CD56bright cells, NK CD56dim cells, DC, B cells, Th1 cells, cytotoxic cells, neutrophils, iDC, T cells, pDC, macrophages, Treg, and aDC cells (Figure 5(b)). In addition, the different expression of 24 types of infiltrating immune cells between LINC02249 high and low expression subgroups was shown in Figure 5(c). According to these findings, an increase in LINC02249 expression might be followed by a decrease in anticancer immune infiltration, which would lead to a poorer prognosis for patient survival.

## 4. Discussion

SKCM is caused by the change of skin melanocytes into cancerous cells [25]. It is characterized by a high degree of malignancy, a strong invasiveness, and the fact that it can affect people of any age [26, 27]. If it is not actively treated, there is a high risk that it will migrate through the dermis and metastasis. Patients diagnosed with SKCM have a bad prognosis and a high mortality rate as a result [28]. Even though a number of different treatment modalities like targeted therapy, immunotherapy, chemotherapy, and radiotherapy have been utilized to enhance long-term survivals of patients, issues like limited drug sensitivity and drug resistance still need to be taken into consideration [29, 30]. Therefore, it is of the utmost need to locate additional prognostic biomarkers and possible medications for SKCM.

Over the past few years, an increasing number of studies have indicated that long noncoding RNAs (lncRNAs) may be implicated in the advancement of SKCM. For instance, An et al. reported that AGAP2-AS1 was significantly higher in melanoma than in healthy tissues, and the researchers found that the level of AGAP2-AS1 in cancer tissues was significantly connected to the TNM stage of the patient's malignancy. Individuals who had a high level of AGAP2-AS1 had a survival duration that was noticeably lower than that of patients who had a low level of AGAP2-AS1, and this was true for both progression-free survival and overall survival [31]. In melanoma, inhibiting the expression of the long noncoding RNA AGAP2-AS1 has the functional effect of reducing carcinogenesis and ferroptosis resistance via the SLC7A11-IGF2BP2 pathway. Shan et al. showed that lncRNA SNHG8 levels were distinctly increased in melanoma specimens, and melanoma cell viability, migration, and invasion were all inhibited by inhibiting the lncRNA SNHG8 pathway, which was mediated by the miRNA-656-3p/SERPINE1 axis [32]. We initially found the differential expression of LINC02249 in SKCM utilizing data on pan-cancer that was freely available to the public in order to acquire a comprehensive grasp of the role that LINC02249 plays in SKCM. We were able to demonstrate that LINC02249 was differentially expressed in a number of different cancers; in particular, the expression of LINC02249 was found to be considerably elevated in SKCM in comparison to other tumors. According to our findings, the differential expression of LINC02249 might be exclusive to certain tissues, and it served as a tumor promotor in SKCM. Then, KEGG assays confirmed that LINC02249 may play an important role in the regulation of PPAR signaling pathway, viral protein interaction with cytokine and cytokine receptor, cellular senescence, oocyte meiosis, and cell cycle. Moreover, we analyzed the prognostic value of LINC02249 expression in SKCM patients, finding that high LINC02249 expression was associated with shorter overall survival and disease-specific survival. More importantly, multivariate analysis showed that LINC02249 were an independent prognostic factor for overall survival and disease-specific survival of SKCM patients.

We explored the expression of LINC02249 and the infiltration of immune cells in the tumor tissue because gene alterations may contribute to an abnormal immune microenvironment in malignancies [33, 34]. LINC02249 is a lncRNA that may lead to abnormal immune microenvironments in cancers. Immune evasion and immunosuppression have emerged as critical areas for tumor-targeted therapy in recent years [35, 36]. The development of tumor antigen-specific T cells is a crucial step in the process of antitumor immune surveillance, and macrophages have a part to play at every stage of the progression of tumors, from tumor initiation through tumor spread. It is beyond question that macrophages with an M2-like phenotype might contribute to immunosuppression, tumor development, and angiogenesis [37, 38]. In our study, we found the expression of LINC02249 was distinctly negatively associated with stromal scores, immune scores, and estimate scores. Moreover, we found that the results showed that among 24 types of infiltrating immune cells, 11 types showed a strong negative correlation with NK CD56bright cells, NK CD56dim cells, DC, B cells, Th1 cells, cytotoxic cells, neutrophils, iDC, T cells, pDC, macrophages, Treg, and aDC. Incorrect signal transduction had a significant role in the progression of the tumor. T cell infiltration and its functional pathway is a signaling pathway that has been conserved throughout the evolution. This pathway regulated the immunologic status of the tumor, and as a result, has an effect on the outcome. Our new knowledge of how LINC02249 influenced the immune microenvironment in individuals with SKCM may be beneficial to the development of future medicines that target tumors specifically.

This study had several limitations. First, the information used in our research came from publicly available sources, such as the TCGA datasets. Thus, an evaluation of the reliability of the data was impossible. Second, extensive research must be conducted on the biological roles of LINC02249, and these functions must be clarified through *in vitro* and *in vivo* tests. Particular attention must be paid to the immunological infiltration process.

## 5. Conclusion

We observed that LINC02249 was significantly expressed in a variety of human cancers (including SKCM), and its upregulation was related with a poor outcome in SKCM. Besides, we discovered that the expression of LINC02249 was connected to the invasion of immune cells. LINC02249 has the potential to function as an independent prognostic biomarker for SKCM. In addition, the research lays the theoretical groundwork for treatment objectives.

## Figures and Tables

**Table 1:** Association of LINC02249 expression levels with clinical factors in SKCM patients.

Characteristics	Low expression of LINC02249	High expression of LINC02249	*p*
*n*	235	236	

Age, *n* (%)			0.200
≤60	132 (28.5%)	120 (25.9%)	
>60	97 (21%)	114 (24.6%)	

Gender, *n* (%)			0.063
Female	79 (16.8%)	100 (21.2%)	
Male	156 (33.1%)	136 (28.9%)	

Pathologic stage, *n* (%)			0.061
Stage I	46 (11.2%)	31 (7.5%)	
Stage II	57 (13.8%)	83 (20.1%)	
Stage III	84 (20.4%)	87 (21.1%)	
Stage IV	11 (2.7%)	13 (3.2%)	

Radiation therapy, *n* (%)			0.120
No	183 (39.4%)	200 (43.1%)	
Yes	47 (10.1%)	34 (7.3%)	

Age, median (IQR)	57 (47, 68)	60 (48, 73)	0.040

**Table 2:** Univariate and multivariate analyses for overall survival in SKCM patients.

Characteristics	Total (*N*)	Univariate analysis		Multivariate analysis	
		Hazard ratio (95% CI)	*p* value	Hazard ratio (95% CI)	*p* value
Pathologic stage	410				
Stage I and Stage II	217	Reference			
Stage III and Stage IV	193	1.617 (1.207–2.165)	**0.001**	1.723 (1.283–2.314)	**<0.001**

Gender	456				
Female	172	Reference			
Male	284	1.172 (0.879–1.563)	0.281		

Age	456				
≤60	246	Reference			
>60	210	1.656 (1.251–2.192)	**<0.001**	1.461 (1.089–1.962)	**0.012**

LINC02249	456				
Low	226	Reference			
High	230	1.724 (1.315–2.260)	**<0.001**	1.693 (1.267–2.263)	**<0.001**

**Table 3:** Univariate and multivariate analyses for disease-specific survival in SKCM patients.

Characteristics	Total (*N*)	Univariate analysis		Multivariate analysis	
		Hazard ratio (95% CI)	*p* value	Hazard ratio (95% CI)	*p* value
Pathologic stage	405				
Stage I and Stage II	215	Reference			
Stage III and Stage IV	190	1.536 (1.125–2.096)	**0.007**	1.632 (1.192–2.233)	**0.002**

Gender	450				
Female	172	Reference			
Male	278	1.161 (0.855–1.575)	0.340		

Age	450				
≤60	244	Reference			
>60	206	1.699 (1.258–2.294)	**<0.001**	1.484 (1.085–2.031)	**0.014**

LINC02249	450				
Low	224	Reference			
High	226	1.690 (1.266–2.255)	**<0.001**	1.627 (1.196–2.213)	**0.002**

## Data Availability

The datasets used to support the findings of this study are available from the corresponding author upon reasonable request.

## Supplementary Materials

Supplementary Materials Table S1: the full names of pan-cancers in TCGA datasets.
